# Emergency department visits and boarding for pediatric patients with suicidality before and during the COVID-19 pandemic

**DOI:** 10.1371/journal.pone.0286035

**Published:** 2023-11-01

**Authors:** Amy R. Zipursky, Karen L. Olson, Louisa Bode, Alon Geva, James Jones, Kenneth D. Mandl, Andrew McMurry

**Affiliations:** 1 Computational Health Informatics Program, Boston Children’s Hospital, Boston, Massachusetts, United States of America; 2 Department of Pediatrics, Harvard Medical School, Boston, Massachusetts, United States of America; 3 Division of Emergency Medicine, Boston Children’s Hospital, Boston, Massachusetts, United States of America; 4 Peter L. Reichertz Institute for Medical Informatics of TU Braunschweig and Hannover Medical School, Hannover, Lower Saxony, Germany; 5 Division of Critical Care Medicine, Department of Anesthesiology, Critical Care, and Pain Medicine, Boston Children’s Hospital, Boston, Massachusetts, United States of America; 6 Department of Biomedical Informatics, Harvard Medical School, Boston, Massachusetts, United States of America; Emory University School of Medicine, UNITED STATES

## Abstract

**Objective:**

To quantify the increase in pediatric patients presenting to the emergency department with suicidality before and during the COVID-19 pandemic, and the subsequent impact on emergency department length of stay and boarding.

**Methods:**

This retrospective cohort study from June 1, 2016, to October 31, 2022, identified patients ages 6 to 21 presenting to the emergency department at a pediatric academic medical center with suicidality using ICD-10 codes. Number of emergency department encounters for suicidality, demographic characteristics of patients with suicidality, and emergency department length of stay were compared before and during the COVID-19 pandemic. Unobserved components models were used to describe monthly counts of emergency department encounters for suicidality.

**Results:**

There were 179,736 patient encounters to the emergency department during the study period, 6,215 (3.5%) for suicidality. There were, on average, more encounters for suicidality each month during the COVID-19 pandemic than before the COVID-19 pandemic. A time series unobserved components model demonstrated a temporary drop of 32.7 encounters for suicidality in April and May of 2020 (p<0.001), followed by a sustained increase of 31.2 encounters starting in July 2020 (p = 0.003). The average length of stay for patients that boarded in the emergency department with a diagnosis of suicidality was 37.4 hours longer during the COVID-19 pandemic compared to before the COVID-19 pandemic (p<0.001).

**Conclusions:**

The number of encounters for suicidality among pediatric patients and the emergency department length of stay for psychiatry boarders has increased during the COVID-19 pandemic. There is a need for acute care mental health services and solutions to emergency department capacity issues.

## Introduction

Suicide was the second leading cause of death for 10- to 14-year-olds and the third leading cause of death for 15- to 19-year-olds in 2021 [1]. Since the start of the COVID-19 pandemic [2–8], there has been a documented increase in suicidal ideation and attempt among pediatric patients. This has resulted in an increase in emergency department (ED) visits [7, 9] further straining EDs in the United States [10].

Patients visiting the ED for mental health issues are twice as likely to be hospitalized compared to other patients [11]. Because there is a shortage of inpatient psychiatric beds and limited outpatient mental health services, patients presenting to the ED for mental health reasons often board in the ED for long periods of time [10, 12, 13]. A review of pediatric mental health boarding in 2019 noted that 23 to 58% of patients with mental or behavioral health concerns boarded in the ED and that average boarding duration in the ED was five to 41 hours [13]. This problem has been exacerbated during the COVID-19 pandemic, with increased waiting times for pediatric psychiatric beds [14]. A survey of pediatric hospitalists inquiring about boarding in the ED and inpatient setting in the United States and Canada found that 75% of those that responded noted an increase in boarding duration and 84% an increase in boarding frequency during the COVID-19 pandemic [12]. Patients boarding in the ED often do not have access to the psychiatric services they need for optimal care [12]. The urgent need for solutions to ED boarding and mental health capacity issues has been recognized [10, 15]. Many studies to date have focused on the first year of the pandemic, and have not examined boarding and capacity issues. We sought to quantify the increase in pediatric patients presenting to the ED with suicidality during the COVID-19 pandemic and the subsequent impact on ED length of stay (LOS) and boarding. We additionally sought to describe the population of pediatric patients presenting to the ED with suicidality before and during the COVID-19 pandemic.

## Methods

This is a retrospective cohort study using the electronic health records (EHR) of patients who presented to the ED at Boston Children’s Hospital (BCH), a pediatric academic medical center, between June 1, 2016 and October 31, 2022. Patients between 6 and 21 years old were included and age was recorded as an integer [16]. Patients with multiple ED visits were counted once for each visit and could be included in different age groups over the course of the study. The study received exempt status from the Institutional Review Board at BCH.

Suicidality is defined using International Classification of Diseases, 10th Revision (ICD-10) codes selected from those used in prior studies [17–25]. We included discharge diagnosis and billing codes for suicidal ideation (R45.851), suicide attempt (T14.91*), and codes for intentional self-harm (S1 Table in S1 File). Codes include all those listed in the Suicide and Self-harm group of the Child and Adolescent Mental Health Disorders Classification System (CAMHD-CS) from the Children’s Hospital Association [26]. All patients 12 years of age or older presenting to the ED were screened for suicidal ideation using the Ask Suicide-Screening Questions (ASQ) Toolkit [27].

ED LOS is defined as the time between ED check-in and check-out. Prolonged LOS was defined as more than 24 hours.

Psychiatry boarders are patients who remained in the ED for 24 hours or more and/or needed a higher level of psychiatric care (i.e. inpatient psychiatric services) before discharge from the ED. They were identified by the existence of a standard psychiatry boarder form in the EHR that was completed by a psychiatry resource specialist after the patient was assessed by the emergency psychiatric service. Some patients remained in the ED awaiting inpatient psychiatric care and others were transferred to a medical ward awaiting inpatient psychiatric care depending on inpatient bed availability. This study focused solely on boarding time in the ED and did not examine boarding time spent on a medical ward.

COVID-19 pandemic periods were defined as pre-COVID-19 (June 1, 2016 to February 29, 2020) and COVID-19 (March 1, 2020 to October 31, 2022) [28].

Race and ethnicity were self-reported by patients or their parents or guardians at intake. They could select one or more racial categories including other and indicate their ethnicity as Hispanic or Latino if applicable. For this study, race and ethnicity were coded as mutually exclusive categories: Hispanic/Latino (any race group), and Non-Hispanic/Latino groups: Asian, Black/African American, Multiracial, Other Race, White, and a final category for Unknown.

Preferred language was self-reported by patients or their parents or guardians at intake. For this study, responses were coded into four mutually exclusive categories: English, Spanish, Other language, and Unknown.

Seasons were defined as winter (January, February, March), spring (April, May, June), summer (July, August, September), and fall (October, November, December). Seasonality often characterizes data associated with calendar dates. A recent study of pediatric mental health related ED visits from 2016 to 2021 showed that fewer visits occurred in July and August, except during 2020 [29].

### Analysis

The number of ED encounters with ICD-10 codes for suicidal ideation, suicide attempt, and intentional self-harm pre-COVID-19 and during COVID-19 were compared. We also compared the demographic characteristics of patients during these two time periods. ED LOS for encounters with suicidality diagnoses were compared for those with and without psychiatry boarders during the two time periods. Analyses used Chi-square and analysis of variance (ANOVA) with post-hoc Tukey tests. Least squares means (LSM) and 95% confidence limits (CL) or differences between LSMs are reported for ANOVA results. Analyses were performed using SAS 9.4 (SAS Institute Inc., Cary, NC).

Unobserved components models (UCM) were used to describe monthly counts of ED encounters for suicidality. Time series can be described in terms of their components such as trend, seasonality, and cycles, and can include regression effects [30–33]. Our initial model included components for trend and seasonality. Regression variables were added to assess the impact of COVID-19 in 2020, with April 2020 as the initial month of impact. Statistical change point analysis was used to evaluate other notable trend changes. Analyses were performed using the UCM Procedure in SAS 9.4. P values less than 0.05 were considered significant.

## Results

There were 179,736 ED encounters during the study period and 6,215 (3.5%) had an ICD-10 code for suicidality. ANOVA revealed main effects for the COVID-19 pandemic period and season (p<0.001 for each) and no significant interaction. On average, there were more suicidality encounters each month during the COVID-19 time period (LSM 101.8, CL95.2, 108.4) than during the pre-COVID-19 time period (LSM 67.3, CL 61.8, 72.8). Compared to summer months, on average there were 19.2 (CL 3.7, 34.7) more suicidality encounters during spring, 30.6 (CL 14.9, 46.4) more during fall, and 33.3 (CL 17.4, 49.2) more during winter months.

Among all ED encounters, we examined how many suicidality encounters versus all others were characterized by a prolonged LOS or a psychiatry boarder form in the EHR. More suicidality encounters (2,916, 46.9%) had a prolonged ED LOS than encounters without that diagnosis (2,616, 1.5%, p<0.001). More suicidality encounters had a completed boarder form (4,723, 76.0%) than other ED encounters (4,701, 2.7%, p<0.001).

The demographic characteristics of patients with a suicidality diagnosis during ED encounters in the pre-COVID-19 and COVID-19 time periods are shown in Table 1. The majority of encounters (84.7%) were with patients 12–18 years old. Differences between the COVID-19 pandemic periods were not significant (p = 0.600). The majority of patients were female, and they comprised a greater percentage of patients during the COVID-19 time period (71.6%) than during the pre-COVID-19 time period (65.4%, p<0.001). Race and ethnicity was reported as White, non-Hispanic for the majority of patients (52.6%) during suicidality encounters, followed by Hispanic/Latino (14.9%) and Black/African American, non-Hispanic (12.4%). The distribution of categories varied somewhat between the time periods (p<0.001). Post-hoc analyses indicated that one category, Unknown, differed by pandemic period. English was reported as the preferred language for the majority of encounters (91.4%), followed by Spanish (4.8%), and other languages (2.5%). Post-hoc analyses indicated that one category, Unknown, differed by pandemic period.

**Table 1:** Characteristics of patients with a suicidality diagnosis during ED encounters before and during the COVID-19 pandemic.

Variable	Values	Pre-COVID-19 N 3,013		COVID-19 N 3,202		
		Number	Percent	Number	Percent	p-value
**Age (years)**	6–11	364	12.1	361	11.3	0.600
	12–18	2,537	84.2	2,724	85.1	
	19–21	112	3.7	117	3.7	
**Sex**	Female	1,970	65.4	2,292	71.6	<0.001
	Male	1,043	34.6	910	28.4	
**Race and ethnicity** ^**a**^	Asian, non-Hispanic	126	4.2	113	3.5	<0.001
	Black/African American, non-Hispanic	402	13.3	367	11.5	
	Hispanic/Latino	458	15.2	471	14.7	
	Multiracial, non-Hispanic	51	1.7	65	2.0	
	Other race, non-Hispanic	211	7.0	182	5.7	
	White, non-Hispanic	1,585	52.6	1,686	52.7	
	Unknown ^b^	180	6.0	318	9.9	
**Preferred language**	English	2,736	90.8	2,945	92.0	0.007
	Spanish	149	4.9	152	4.7	
	Other language	75	2.5	80	2.5	
	Unknown ^b^	53	1.8	25	0.8	

^a^ Categories are mutually exclusive.

^b^ Significant difference between pre-COVID-19 and COVID-19 time periods in post-hoc analyses.

Suicide attempt was coded for 322 encounters (5.2%) and these encounters could also include codes for intentional self-harm (126), ideation (62), or both (57). Intentional self-harm codes, without an attempt code, described 1,016 (16.3%) encounters, 456 with an ideation code. Ideation without attempt or intentional self-harm codes described the majority (4,877, 78.5%) of encounters. There were no differences by time period (p = 0.103).

The self-harm diagnosis codes were grouped into injury mechanism categories from the Centers for Disease Control external cause matrix for intentional self-harm [34]. The majority of the 1,199 encounters with intentional self-harm ICD-10 codes observed in our data were from the “Poisoning, Drug” category (75.8%), followed by “Cut/Pierce” (12.2%). Each of the eight injury categories are listed in Table 2, along with the 28 3-character ICD values that describe the self-harm diagnoses (also see S2-S4 Tables in S1 File).

**Table 2:** Mechanism of injury and diagnosis categories for emergency department encounters with intentional self-harm diagnoses.

CDC Injury Mechanism Number (percent)	ICD3 Rank	ICD3	Encounters (1,199)		Description
			Number	Percent	
Poisoning, Drug 909 (75.8%)	1	T39	393	32.8	Nonopioid analgesics, antipyretics, antirheumatics
	2	T43	292	24.4	Psychotropic NEC
	3	T50	241	20.1	Diuretics and other unspecified
	6	T45	116	9.7	Systemic hematological agents NEC
	7	T42	100	8.3	Antiepileptic, sedative, antiparkinson
	8	T46	61	5.1	Drugs for cardiovascular system
	9	T40	36	3.0	Narcotics, hallucinogens
	10	T44	34	2.8	Drugs for autonomic nervous system
	11	T48	31	2.6	Drugs for smooth and skeletal muscles and respiratory system
	12	T38	27	2.3	Hormones, synthetic substitutes
	17	T47	15	1.3	Drugs for gastrointestinal system
	18	T36	11	0.9	Systemic antibiotics
	19.5	T37	7	0.6	Anti-infectives, antiparasitics
	19.5	T49	7	0.6	Topical agents
Cut/Pierce 146 (12.2%)	4	X78	146	12.2	Intentional self-harm by sharp object
Other specified 118 (9.8%)	5	X83.8^a^	118	9.8	Intentional self-harm by other specified means NEC
Poisoning, Non-drug 51 (4.3%)	14	T51	19	1.6	Toxic effect—alcohol
	16	T65	16	1.3	Toxic effect—other and unspecified substances
	21	T55	5	0.4	Toxic effect—soaps and detergents
	22	T56	4	0.3	Toxic effect—metals
	23.5	T52	3	0.3	Toxic effect—organic solvents
	23.5	T59	3	0.3	Toxic effect—other gases, fumes, vapors
	26.5	T57	1	0.1	Toxic effect—other inorganic substances
	26.5	T60	1	0.1	Toxic effect—pesticides
Suffocation 24 (2%)	13	T71	24	2.0	Asphyxiation
Fire/Burn 17 (1.4%)	15	T54	17	1.4	Toxic effect—corrosive substances
Fall 1 (0.08%)	26.5	X80	1	0.1	Intentional self-harm by jump from high place
Natural/Environmental 1 (0.08%)	26.5	X83.2^a^	1	0.1	Intentional self-harm by exposure to extremes of cold

CDC = Centers for Disease Control and Prevention, ICD3 = International Classification of Diseases (first three characters)

^a^ Fourth character added to X83 to place code into the appropriate injury mechanism category.

The effect of psychiatry boarder status and COVID-19 pandemic period on ED LOS is shown in Table 3. ANOVA revealed significant main effects for boarder status and time period, and a significant interaction (p<0.001 for all). Encounters without psychiatry boarders were not prolonged and post-hoc analyses showed that ED LOS did not differ between the pre-COVID-19 and COVID-19 time periods. However, encounters with boarders were, on average, prolonged and those during the COVID-19 pandemic were 37.4 hours longer than before the COVID-19 pandemic. Also, before the COVID-19 pandemic, suicidality encounters where the patient was boarding were 22.9 hours longer than encounters without boarding. During the COVID-19 pandemic, this difference was 61.7 hours.

**Table 3:** The effect of psychiatry boarder status and COVID-19 pandemic period on ED LOS for patients with a suicidality diagnosis.

COVID-19 Pandemic Period	Psychiatry boarder		ED Length of stay (hours)		
		N	Least squares mean	95% Confidence limits	
**Pre-COVID-19**	No	921	10.0 ^a^	6.7	13.4
**Pre-COVID-19**	Yes	2,092	32.1 ^b^	29.9	34.3
**COVID-19**	No	571	7.8 ^a^	3.6	12.0
**COVID-19**	Yes	2,631	69.4 ^c^	67.5	71.4

In post-hoc analyses, least squares mean values with the same superscript did not differ.

**Fig 1:**
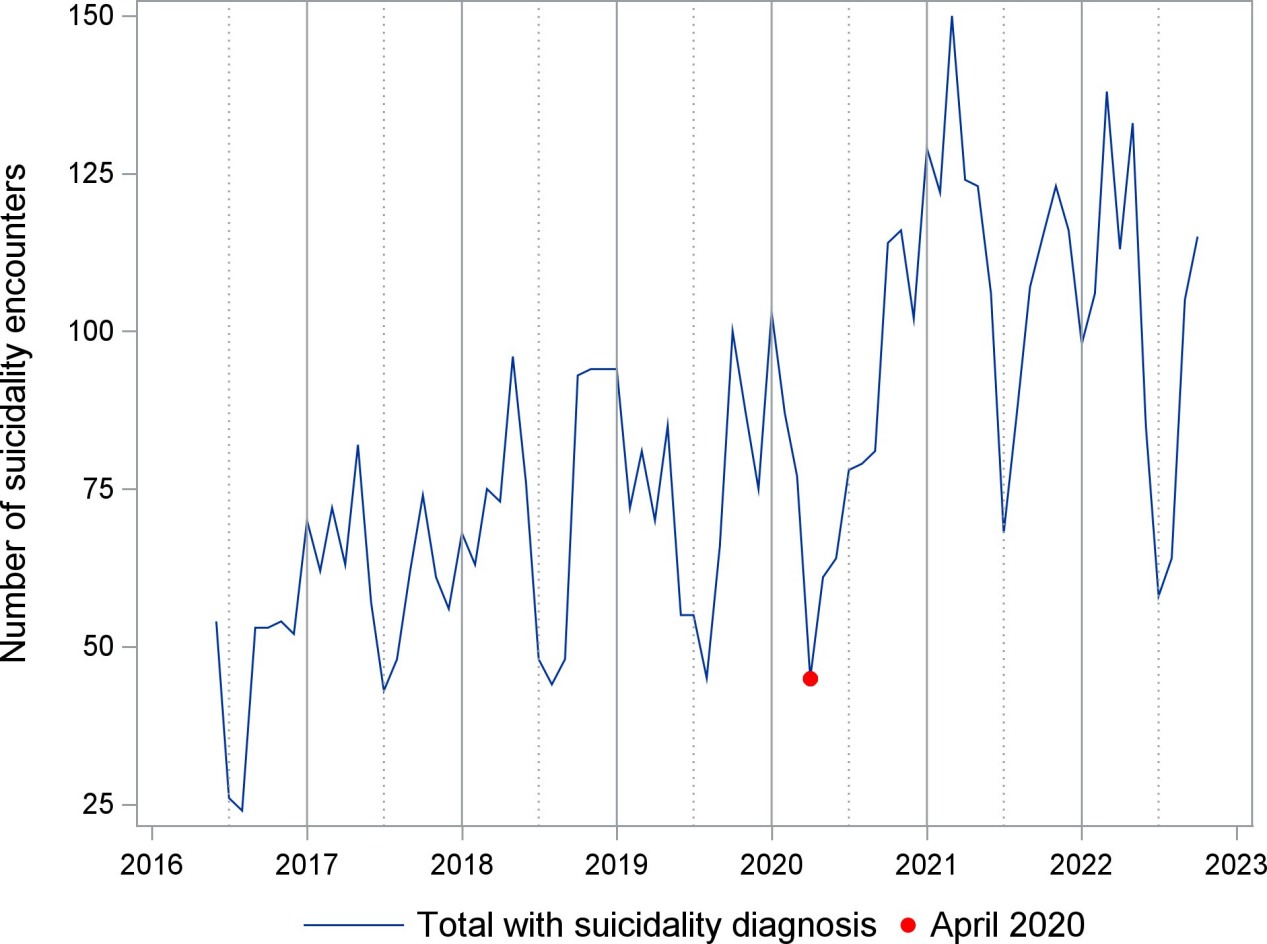
Time series for number of emergency department encounters with suicidality. Number of encounters per month between June 1, 2016 and October 31, 2022.

**Fig 2:**
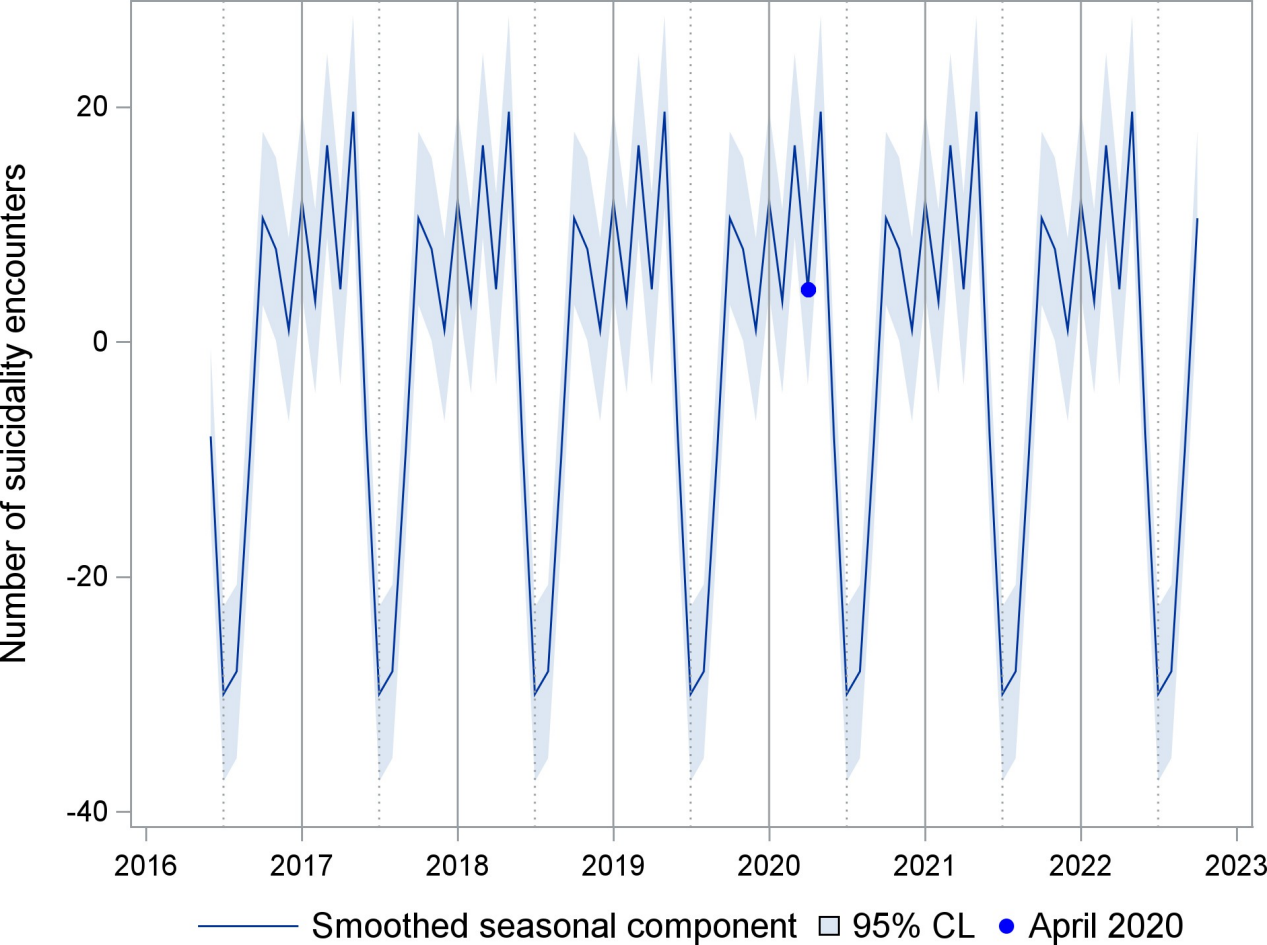
Smoothed seasonal component for emergency department encounters with suicidality. CL = Confidence limits. Seasonal component of a time series unobserved components model.

**Fig 3:**
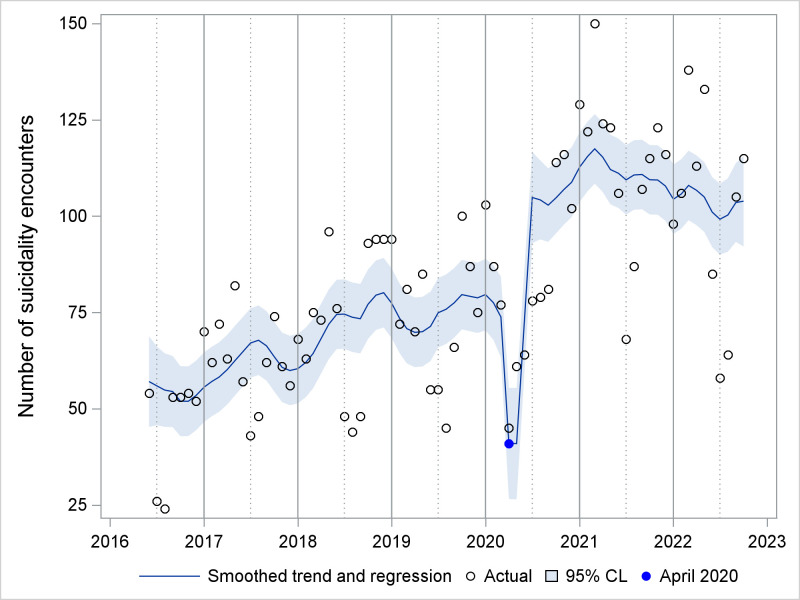
Smoothed trend and regression effects (April-May 2020 drop, July 2020 level shift). CL = Confidence limits. Sum of effects from a time series unobserved components model for emergency department encounters with suicidality.

**Fig 4:**
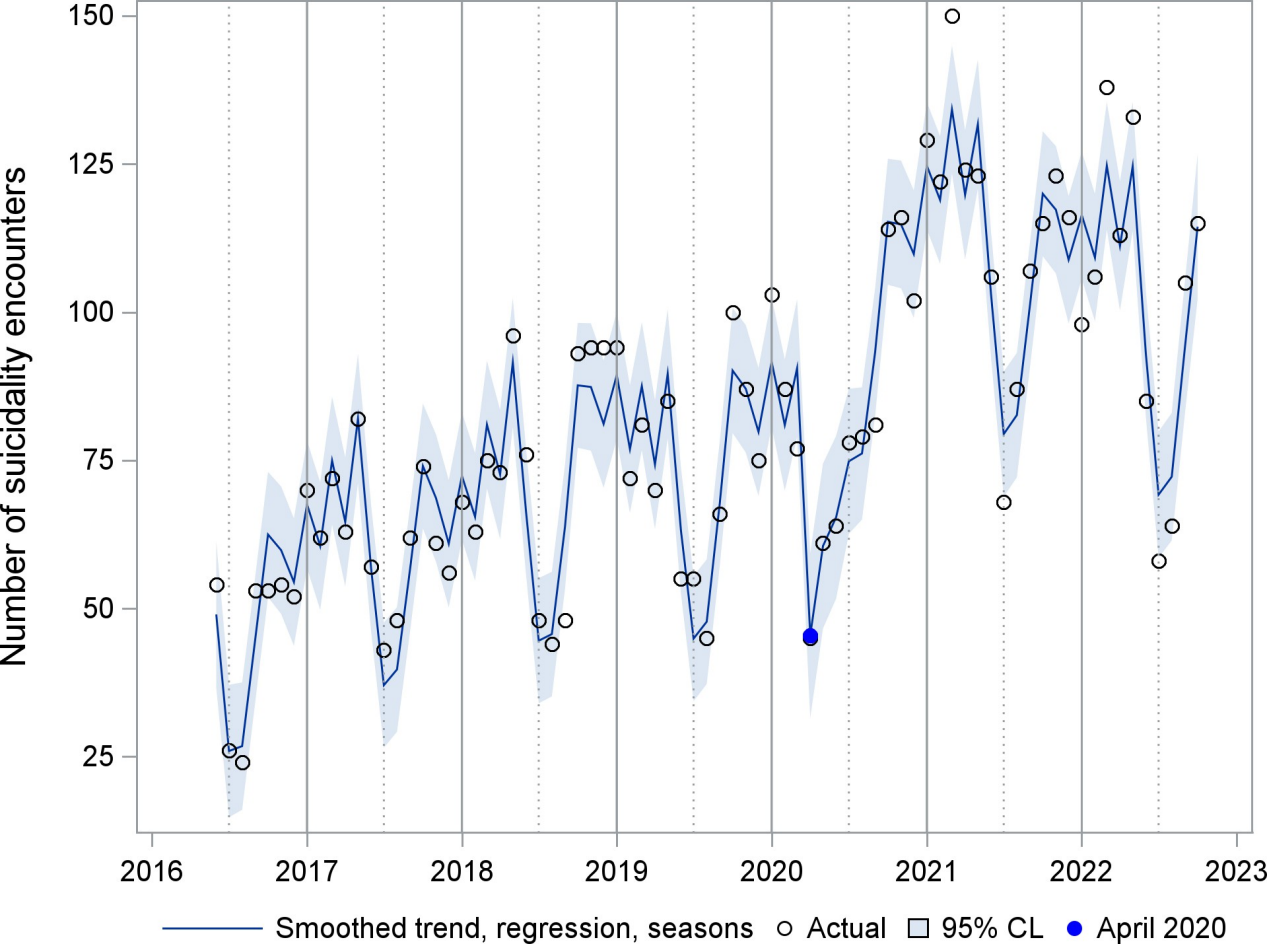
UCM sum of the smoothed trend, regression, and season effects. CL = Confidence limits. Sum of all components except the irregular term of a time series unobserved components model for emergency department encounters with suicidality. Regression effects are a temporary drop in April and May 2020 and a level shift beginning in July 2020.

A time series of the number of ED suicidality encounters is shown in Fig 1. Seasonality is apparent, with lowest points in summer months, except during 2020. The UCM analysis for suicidality encounters included a trend component modeled as a random walk with drift, deterministic seasonality, and two intervention variables. A smoothed plot of the seasonality component is shown in Fig 2. The estimate for level at its final state was 72.7 encounters (standard error (se) 12.2, p<0.001), while the slope was close to zero (0.2, se 0.6, p = 0.711). The trend was effectively a random walk with little or no drift. Two intervention variables described the impact of COVID-19. A temporary change lasting two months (April-May 2020) had an effect size of -32.7 encounters (se 9.5, p<0.001). A level change beginning in July 2020 had a sustained effect size of 31.2 (se 10.6, p = 0.003). Fig 3 shows the smoothed sum of the trend and regression effects (April-May 2020 drop, increase since July 2020) by isolating these effects from the complete series. A smoothed plot of the sum of all the components and regression effects, minus the irregular term, is shown in Fig 4.

## Discussion

After an initial drop at the beginning of the COVID-19 pandemic in April and May 2020, the number of ED encounters for suicidality increased during the COVID-19 pandemic.

Previous studies have shown a decrease in ED visits overall and ED visits for psychiatric issues early in the COVID-19 pandemic [6, 35, 36]. Individuals may have accessed care less frequently early in the COVID-19 pandemic due to fear of contracting COVID-19. The subsequent increase we observed in ED visits for suicidality is consistent with previous literature. Existing literature, mainly focusing on data up to 2021 [2, 4–7, 14], found an increase in pediatric suicidality during the COVID-19 pandemic as well as increased ED visits [2–7, 14] and hospitalizations [37]. A number of factors likely contributed to the increase in presentations for suicidality. Early in the pandemic, with stay-at-home orders, there was decreased access to mental health services including school services [38]. With limited access to services, more patients may have had worsening mental health symptoms leading to suicidality. Persistent stressors related to COVID-19 may have resulted in increased suicidality among pediatric patients. The increased presentations could be a result of delayed presentations from early in the COVID-19 pandemic. Our results also describe an increase in the number and duration of ED boarding for those presenting to the ED with suicidality. Previous literature has described an increase in mental health boarding in general through 2021 and has included ED and inpatient unit stays or other temporary location stays in the definition of boarding [12, 14]. This study specifically highlights capacity issues related to the ED and patients presenting with suicidality. Furthermore, with data through October 2022, our study demonstrates the sustained impact of COVID-19 on pediatric suicidality and the need for improved care for patients presenting with suicidality. While solutions to these issues are complex, previous literature has explored possible approaches. To reduce ED capacity, previous studies have supported efficacy of initiatives during ED visits such as suicide prevention interventions [39] and improved primary care provider follow-up [40]. Literature has proposed psychosocial interventions during boarding including psychoeducation, behavioral activation, and relaxation training [41].

This study has some limitations. First, while we chose diagnostic codes used in the published literature to identify patients with suicidality [17–25], these ICD-10 codes are nonetheless intended for patient billing. Consistent with previous literature, we included codes for suicide and for intentional self-harm. We recognize the challenge clinically in deciphering whether self-harm is intentional and whether the intention of the patient was to die; this may have resulted in an overestimation of suicidality in our study. Second, though our time series demonstrates a compelling trend, other secular changes during the COVID-19 pandemic may not be accounted for. Pediatric primary care was limited at points during the COVID-19 pandemic due to factors such as access to personal protective equipment and ability to isolate infectious patients [42]. This may have led patients to present to the ED rather than accessing the primary care settings that they would have prior to the COVID-19 pandemic. Finally, our study was in a large, well-resourced children’s hospital in an urban setting. While studies conducted in other settings have found comparable results [2, 4–7, 35], these findings may not generalize to all settings.

Overall, we demonstrate an increase in patients with suicidality that presented to the ED during the COVID-19 pandemic and the capacity issues facing the ED. Future studies should focus on expanding this work to multiple medical centers. This work supports the need for an increase in acute care mental health services and solutions to ED capacity issues.

## Supporting information

S1 File Contains all supporting tables and data files. (XLSX)
